# A Modified RNA-Seq Approach for Whole Genome Sequencing of RNA Viruses from Faecal and Blood Samples

**DOI:** 10.1371/journal.pone.0066129

**Published:** 2013-06-10

**Authors:** Elizabeth M. Batty, T. H. Nicholas Wong, Amy Trebes, Karène Argoud, Moustafa Attar, David Buck, Camilla L. C. Ip, Tanya Golubchik, Madeleine Cule, Rory Bowden, Charis Manganis, Paul Klenerman, Eleanor Barnes, A. Sarah Walker, David H. Wyllie, Daniel J. Wilson, Kate E. Dingle, Tim E. A. Peto, Derrick W. Crook, Paolo Piazza

**Affiliations:** 1 Wellcome Trust Centre for Human Genetics, Oxford, United Kingdom; 2 Nuffield Department of Medicine, University of Oxford, John Radcliffe Hospital, Oxford, United Kingdom; 3 Oxford NIHR Biomedical Research Centre, John Radcliffe Hospital, Oxford, United Kingdom; 4 Department of Statistics, University of Oxford, Oxford, United Kingdom; 5 Nuffield Department of Clinical Laboratory Sciences, Headley Way, University of Oxford, John Radcliffe Hospital, Oxford, United Kingdom; Columbia University, United States of America

## Abstract

To date, very large scale sequencing of many clinically important RNA viruses has been complicated by their high population molecular variation, which creates challenges for polymerase chain reaction and sequencing primer design. Many RNA viruses are also difficult or currently not possible to culture, severely limiting the amount and purity of available starting material. Here, we describe a simple, novel, high-throughput approach to Norovirus and Hepatitis C virus whole genome sequence determination based on RNA shotgun sequencing (also known as RNA-Seq). We demonstrate the effectiveness of this method by sequencing three Norovirus samples from faeces and two Hepatitis C virus samples from blood, on an Illumina MiSeq benchtop sequencer. More than 97% of reference genomes were recovered. Compared with Sanger sequencing, our method had no nucleotide differences in 14,019 nucleotides (nt) for Noroviruses (from a total of 2 Norovirus genomes obtained with Sanger sequencing), and 8 variants in 9,542 nt for Hepatitis C virus (1 variant per 1,193 nt). The three Norovirus samples had 2, 3, and 2 distinct positions called as heterozygous, while the two Hepatitis C virus samples had 117 and 131 positions called as heterozygous. To confirm that our sample and library preparation could be scaled to true high-throughput, we prepared and sequenced an additional 77 Norovirus samples in a single batch on an Illumina HiSeq 2000 sequencer, recovering >90% of the reference genome in all but one sample. No discrepancies were observed across 118,757 nt compared between Sanger and our custom RNA-Seq method in 16 samples. By generating viral genomic sequences that are not biased by primer-specific amplification or enrichment, this method offers the prospect of large-scale, affordable studies of RNA viruses which could be adapted to routine diagnostic laboratory workflows in the near future, with the potential to directly characterize within-host viral diversity.

## Introduction

Rapid, high-throughput and accurate whole genome sequencing of RNA viral pathogens such as Norovirus and Hepatitis C virus (HCV) has enormous potential for the investigation of local transmission and widespread dispersal [1]. Developing generic methods that yield whole genomic sequence has been challenging because the substantial genomic variation within circulating populations of RNA viruses poses difficulties for primer design [2]. Some RNA viruses are difficult or impossible to grow in tissue culture precluding obtaining highly purified viral nucleic acid in suitable concentrations for whole genome sequencing. The challenges associated with non-culture-based approaches to purifying viral RNA from samples such as faeces and blood that are heavily contaminated by RNA from other sources has severely limited the application of high-throughput sequencing [3]. Unlike whole genome sequencing of bacteria, which is now well developed [4] [5] [6] [7] [8], sequencing of viruses has therefore not benefitted from dramatic advances in sequencing capacity.

Approaches for successfully sequencing RNA viruses have previously been dependent, in some way, on target-specific primer-based amplification of viral genomes [9]. These amplicons can then be sequenced using Sanger sequencing or next-generation sequencing platforms such as Roche 454 and Illumina [10]. Recent examples of this approach include studies of Norovirus evolution [11] and HCV diversity [12]. Next-generation sequencing of amplicons and partial genome fragments from RNA viruses have been used to investigate variants within populations of Human Immunodeficiency Virus and HCV infecting individual patients [13] [14] [15]. An enhancement on this underlying amplification approach uses enrichment of target viral sequences incorporating primers as “bait” to capture larger genomic fragments [16] [17]. All these approaches are expensive, labour-intensive, slow and inflexible, and may require *a priori* knowledge of partial or approximate virus sequence, utilising different primers for different virus strain-groups. In turn, assumptions about the sequences present can bias resulting data, altering the representation of the viral genomic sequence at a population level in a sample [18].

Here we adapt a strategy for high-throughput RNA sequencing for use on RNA viruses present in blood and faeces using RNA shotgun sequencing (RNA-Seq) [19] [20]. We show that this technology can not only generate near whole genome sequences, but can also recover the sequence of multiple within-host variants of highly diverse pathogens such as Norovirus and HCV. This method may be deployed rapidly, and cheaply, using both accessible bench-top and higher capacity platforms. The approach can be used to successfully characterise un-culturable viral genomes as an alternative to a PCR-amplicon based sequencing. We anticipate that this efficient sequencing of variable RNA viruses will bring a step change to both basic and translational research.

## Methods

### Ethics Statement

This study was conducted in compliance with the Data Protection Act (DPA number: Z5886415), and National Health Service research governance. For Norovirus sampling, the Modernising Medical Microbiology study protocol version 1.0 was approved by the Berkshire Research Ethics Committee on the 1st October 2010 (10/H0505/83) and the UK National Information Governance Board (8-05(e)/2010). For HCV samples, ethical approval was approved by the Oxfordshire Research Ethics Committee A on the 15^th^ April 2004, and patients gave written informed consent for these samples to be used prior to sequencing (04.OXA,010).

### Sample Collection and Preparation

#### Faecal samples for Norovirus sequencing

Faecal samples were collected during gastroenteritis outbreaks at the Oxford University Hospitals NHS Trust Hospitals in 2010–2011, as part of the infection service provision of the Trust. Reverse Transcriptase Polymerase Chain Reaction (RT-PCR) [21] was used for initial diagnostic confirmation of Norovirus in all faecal specimens. Viral copy numbers were further determined by quantitative PCR (qPCR) using genogroup specific Taqman probes and primers [22].

#### Blood samples for HCV sequencing

Blood samples were obtained from subtype-3a HCV infected, treatment naive patients with chronic infection. Plasma was obtained by centrifugation and stored within 5 hours of collection at −80°C. Patients were recruited from the Oxford University Hospitals NHS Trust Hospitals.

### Total RNA Isolation

#### Faecal samples (Norovirus)

Total RNA from faecal samples was isolated using the Fujifilm Quickgene DNA tissue kit SII under the manufacturer's RNA extraction from stool protocol for the Fujifilm Quickgene Mini-80 nucleic acid isolation system (Fujifilm Corp., Tokyo, Japan). Three hundred microlitres of supernatant from a 10% clarified emulsion of faeces was used to prepare the lysate. Fifty microlitres of RNA was eluted from the Mini-80 device. The resulting RNA samples were initially quantified by Nanodrop spectrophotometer to estimate concentrations. Samples were stored in −80°C freezers between uses.

#### Plasma samples (HCV)

Plasma was concentrated by high speed centrifugation (23,600× g for 1 h) at 4°C. In order to test whether RNA samples available in archival sources are amenable for high throughput sequencing using a simple workflow, we also used RNA extraction methods currently implemented in many clinical and research laboratories. For HCV, viral RNA was extracted using a QIAmp viral RNA minikit (Qiagen, Hilden, Germany) following the manufacturer's protocol.

### Library Preparation for Sequencing

#### Norovirus amplicon preparation for Sanger sequencing

Reverse transcription and first strand cDNA synthesis were performed using the Accuscript High Fidelity 1st strand cDNA synthesis kit (Agilent, Santa Clara, California, USA) following the manufacturer's published protocol. PCR amplification was then performed using specific in-house primers covering the seven overlapping amplicons of the genome of Norovirus GII.4 (see Table S1), including internal primers to increase depth of coverage using Sanger sequencing. PCR products were then confirmed by agarose gel electrophoresis. The reaction products were separated and detected with a Prism 3730 automated DNA sequencer (Applied Biosystems, Foster City, CA, USA). Sequences were assembled from the resultant chromatograms with the STADEN suite of computer programs [23]. Primer sequences at the 5′ and 3′ termini of the overlapping PCR amplicons were excluded.

#### Hepatitis C Sanger sequencing

One-step reverse transcription and first-round PCR in two reactions amplified a 4-kb and a 7-kb product. Second-round nested PCR reactions used >20 PCR primers in pairs generating 10 overlapping viral genomic fragments each of approximately 1 kb, that were sequenced and aligned manually to a reference sequence as previously described [24].

#### Illumina RNA-Seq Library Preparation

Total RNA quantity and integrity were assessed using Quant-IT RiboGreen RNA Assay Kit (Invitrogen, Carlsbad, CA, USA) and Agilent Tapestation 2200 R6K. Libraries for Illumina sequencing were constructed from 100 ng of total RNA using the NEBNext mRNA Sample Prep Kit 1 (New England Biolabs, Ipswich, MA, USA), following the manufacturers' guidelines with minor modifications: end repair in 50 µl reaction volume (40 µl DNA, 5 µl buffer and 5 µl enzyme), post fragmentation clean-up with 1∶2.8× volume Agencourt Ampure RNAClean XP (Beckman Coulter, Pasadena, CA, USA); post cDNA synthesis clean-up with 1∶1.25× volume Ampure XP Beads; post end repair, A-Tailing and adapter ligation clean-ups with 1∶1.8× volume Ampure XP Beads and post-PCR library clean-ups with 1∶1× volume Ampure XP Beads. Additionally, upon ligation of Illumina Adapters (Multiplexing Sample Preparation Oligonucleotide Kit) each library was size selected with two Ampure Bead steps (firstly, 1∶0.7× volume and secondly, the supernatant from the first bind was taken for a 1∶1.7× volume clean-up), selecting 200–600 bp fragments in 30 µl 10 mM Tris-Cl, pH 8.5. Pre-PCR workflow was partially performed using a Beckman Biomek FX and post-PCR steps were performed using a Beckman Biomek NX^P^ and Biomek 3000.

The following custom primers (25 µM each) were used for the PCR enrichment step:

Multiplex PCR primer 1.0

5′-AATGATACGGCGACCACCGAGATCTACACTCTTTCCCTACACGACGCTCTTCCGATCT-3′

Index primer

5′-CAAGCAGAAGACGGCATACGAGAT[INDEX]CAGTGACTGGAGTTCAGACGTGTGCTCTTCCGATCT-3′

Amplified libraries were analysed for size distribution using the Agilent Tapestation 2200 D1K. Libraries were quantified by quantitative RT-PCR using Agilent qPCR Library Quantification Kit and a Mx3005P instrument (Agilent) and relative volumes were pooled accordingly. Finally, a second quantitative RT-PCR was performed to measure the relative concentration of the pool compared to a previously sequenced mRNA library in order to determine the volume to use for sequencing.

#### Improved Fragmentation Library Preparation for Illumina

To increase the library insert size of the sequenced libraries and to further investigate secondary structures within the Norovirus genome, a modified fragmentation method was developed. The total RNA was fragmented using 2 µl fragmentation buffer diluted 1∶4 in nuclease-free water (fragmentation buffer included in the NEBNext mRNA Library Prep Master Mix Set), incubated at 94°C for 5 minutes, placed immediately to ice and followed by adding 2 µl stop solution (not diluted). Post-fragmentation clean-up through to adapter ligation clean-up were as above. Size selection was omitted to avoid losing longer fragments, and instead, an additional bead clean-up after ligation was performed to remove any remaining adapter dimer prior to PCR amplification.

#### Illumina Amplicon Library Preparation

Amplicons were quantified and quality assessed using Quant-IT Qubit dsDNA High Sensitivity Assay (Invitrogen) and 1% E-gel (Invitrogen) respectively. Fifty nanograms were sheared to 400 bp using Covaris™ DNA Shearing (Woburn, MA, USA) and concentrated using 1∶1× volume Ampure XP Beads. Eluted DNA fragments were then processed through standard library preparation procedures (end repair through to Ampure Bead size selection using the NEBNext DNA Library Prep Master Mix Set for Illumina). Post-reaction clean-ups were 1∶1.8× volume Ampure XP Beads and post-PCR libraries were cleaned with 1∶1× volume Ampure XP. Ten cycles of PCR amplification were performed using custom primers as above, and the final library was pooled with Norovirus positive samples from the pilot study.

#### Sequencing

Multiplex libraries were prepared using barcoded primers and a median insert size of 150 bp (increased to 200 bp with the modified method). The pooled libraries from 3 initial Norovirus and 2 HCV samples were sequenced on an Illumina MiSeq with 150 bp paired end reads following standard Illumina protocols. An average of 0.5 Gb of sequence was produced per sample. A larger pool of 77 Norovirus samples was sequenced on an Illumina HiSeq 2000 with 100 bp paired end reads following standard Illumina protocols.

#### Data analysis

Reference sequences for each organism were obtained from GenBank. For Norovirus the sequences were GII.4, and the Norovirus Hu/GII.4/Orange/NSW001P/2008/AU was used as a reference (accession number GQ845367). This was selected as the most common match to the Sanger sequenced 288 bp RT-PCR amplicon used in the diagnostic RT-PCR stage. For HCV the reference sequence used was the subtype 3a strain (accession number AF046866).

Sequences were mapped to the organism-specific reference using Stampy v1.0.14 [25] with no BWA pre-mapping. Bases and single nucleotide variants (SNVs) were called using the SAMtools “mpileup” command with options ‘-E -M0 -Q30 -q30 -o40 -e20 -h100 -m2 -D -S’ and BCFtools [26]. Sites were filtered to avoid unreliable calls using the following criteria:

- A minimum depth of 5 reads at each position
- A minimum average base quality of 10
- A minimum SNV quality of 25
- At least 75% of reads at the position support the call and the position was called as homozygous

Positions were called as heterozygous based on the SAMtools genotype calls. An evolutionary tree was created using BEAST (Bayesian evolutionary analysis sampling trees) depicting all the full genomic sequences with relatedness. Analysis was performed using BEAST v.1.7.5 combining two random number seed chains (10 million iterations each, saving 1 in 1,000 iterations, with a 1 million iteration burn-in) using: HKY substitution; estimated frequency; strict clock; and constant population size coalescent tree prior. This maximum clade credibility tree was computed using TreeAnnotator v.1.7.5 and plotted with Figtree v.1.4.0. [27].

The sequences reported in this paper have been deposited in the European Nucleotide Archive Sequence Read Archive under study accession number ERP002219. The Sanger sequences used for validation for Norovirus comparison have been submitted to EMBL under accession numbers HF952119-HF952135. For HCV, the Sanger sequences used for comparison were Genbank accession numbers GQ356201 and KC836883.

## Results

### 1.) Proof of principle experiment using the Illumina MiSeq bench-top sequencer: Norovirus and HCV

**Figure 1:**
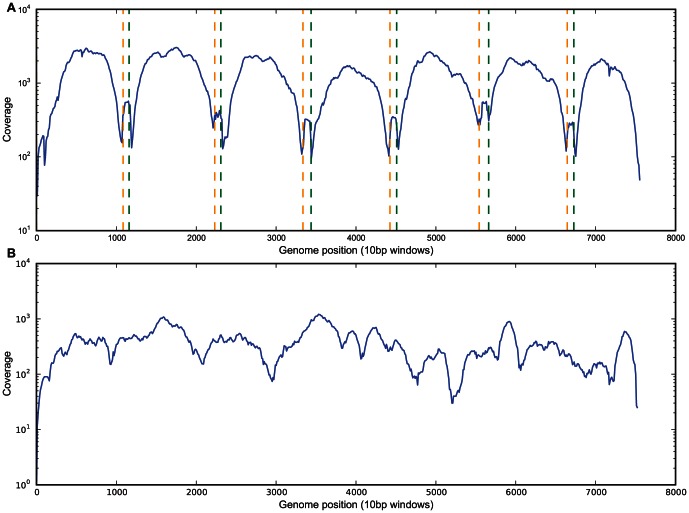
Coverage profiles of one Norovirus sample from amplicon and direct RNA sequencing. A – Coverage across the genome for one Norovirus sample sequenced from PCR amplicons (others similar). Green and orange dotted lined mark the locations of the PCR primers used to generate the amplicons. B – coverage across the genome for the same Norovirus sample sequenced directly from RNA.

**Figure 2:**
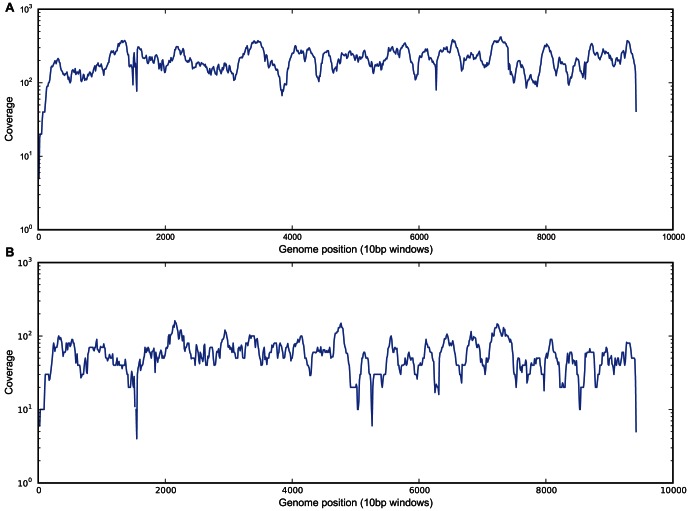
Coverage across the genome for two Hepatitis C samples sequenced directly from RNA.

We chose two RNA viruses for the initial proof of principle study; three faecal samples RT-PCR positive for Norovirus, and two blood samples RT-PCR positive for HCV. For the libraries produced directly from total RNA, 0.12–1.90% (4,188/3,458,332 reads – 50,138/2,671,058 reads) of the total reads mapped to the reference genomes (Table S2), indicating that a low percentage of the total RNA was from the virus of interest. However, despite the low percentage of reads which mapped, sufficient coverage of the virus genome was obtained to achieve a near complete sequence. The coverage of the sequence assembly varied, being lower at the 5′ and 3′ termini of genomes sequenced from cDNA, and at the ends of each of the 7 overlapping PCR amplicons (Figures 1 and 2). This was expected due to the recognised difficulty of recovering suitable short fragments for Illumina sequencing from the ends of DNA molecules [28] [29] [30]. The regions of high and low coverage in the RNA samples were consistent across different genomes from the same organism (Figure 2), which suggest that the amount of coverage obtained is due to an intrinsic property of the viral RNA [31].

Single nucleotide variants (SNVs) were called using SAMtools and BCFtools [26]. After filtering to remove unreliable base calls (see Methods), 97.4–99.1% of the genome could be called in the Norovirus samples, and 97.3–98.9% of the genome in the HCV samples. The majority of the positions which could not be called were clustered at the ends of the genome, and were removed during filtering due to the limited depth of coverage (below 5 reads) and quality at each position.

#### Sanger sequencing for validation

The SNVs called from our Illumina MiSeq sequencing approach were compared with those obtained from Sanger sequencing. For sample 1, 99.1% of the genome could be called by both Sanger and Illumina sequencing, and the base calls by both methods were identical. As the Sanger sequencing for Norovirus was based on amplicon PCR amplification, only partial sequence was obtained for sample 3 since one of the PCR fragments could not be amplified. For this sample, 86.4% of the genome could be called using both methods, and the base calls were all identical. Sample 2 failed repeatedly to produce any PCR products and therefore no Sanger sequence was available for comparison. We had specifically included it in our pilot study to determine whether samples that could not be sequenced by Sanger sequencing could be successfully sequenced using the Illumina platform. Thus overall, for Norovirus we found no differences in a total of 14,019 nt that could be directly compared between Sanger and Illumina custom RNA-Seq sequencing methods. One Norovirus sample (Sample 1) was sequenced twice with Illumina technology, once using a library obtained directly from total RNA (see above) and once from 7 PCR amplicons. Sequences obtained using both methods were identical with the genome generated by Sanger sequencing.

Only one near full-length HCV Sanger sequence was available (sample 4). Due to missing bases at the ends of the genome produced by Sanger sequencing, only 8,051 bp (84% of the genome) could be called by both sequencing methods. The sequences differed at 2 positions. For sample 5, only a partial Sanger sequence (1,491 nt) of the *env* gene was available. Of the 1,491 nucleotides which could be called by both methods (15% of the genome), there were 5 SNV differences and a 1 bp insertion in the Sanger sequence relative to the Illumina sequencing. Thus overall, for HCV we found 8 variants in 9,542 nt that could be directly compared between Sanger and Illumina RNA-Seq sequencing methods, 1 variant per 1,193 nt.

Both viruses yielded heterozygous base calls by our SAMtools-based variant calling pipeline, indicating the presence of within-sample genetic variation. The three Norovirus samples showed 2, 3, and 2 distinct positions called as heterozygous, while the two HCV samples had 117 and 131 positions called as heterozygous. Although we did not investigate these positions further, they indicate the potential to directly characterize within-host viral diversity using Illumina sequencing.

### 2.) Validation using the HiSeq sequencer: Norovirus

To test our method on a larger scale, we sequenced 61 Norovirus patient samples on the HiSeq platform. All samples were positive for Norovirus by RT-PCR prior to sequencing. To determine the reproducibility of our approach, we split 15 larger volume samples into duplicates. Either two libraries were prepared from the same extraction of total RNA (9 pairs), or RNA was extracted from the same faecal sample twice and two separate libraries were prepared (6 pairs). Additionally, one library previously sequenced on the MiSeq platform was also sequenced on the HiSeq (total 77 sequences generated). There was a four to seven month time difference between the extraction and re-extraction from the pairs of samples prepared from the same original source material. Although incidental, this allowed us to ascertain whether the integrity of the faecal material degraded with time. In this total set of 61 patient samples, 16 had full genomes produced by Sanger sequencing, enabling comparison and validation.

Using the HiSeq platform, a mean of 4.6 million reads were produced per sample. The proportion of reads that mapped to the reference genome varied across the samples, ranging from 0.01% to 97.98%; a wider range than seen in the three pilot MiSeq samples (Table S2). Although greater variability would be expected in a larger sample collection, this may also reflect variability in the proportion of the original sample that comprised the viral RNA of interest versus RNA from other sources. We observed a significant correlation between the percentage of reads which mapped to the reference genome and the viral titre estimated by qPCR (rho = 0.4, p<0.0001, Figure S1).

Seventy-six out of 77 sequenced preparations produced sufficient reads to allow 90–98.9% of the reference GII.4 genome bases to be called (mean 97.0%). One sample yielded a low number of reads so that only 4,449 (58.9%) of the reference bases could be successfully called (sample 6). As with the pilot MiSeq study, the bases which could not be called were clustered at the ends of the genome with specific regions consistently yielding low coverage (Figure S2). No differences in the called sequence of 15 duplicate pairs were observed. Of the 12 single samples and 4 pairs of samples where Sanger sequencing was available, the base calls were identical across all samples (total 118,757 nt, 148,421 nt including the 4 repeated samples).

**Figure 3:**
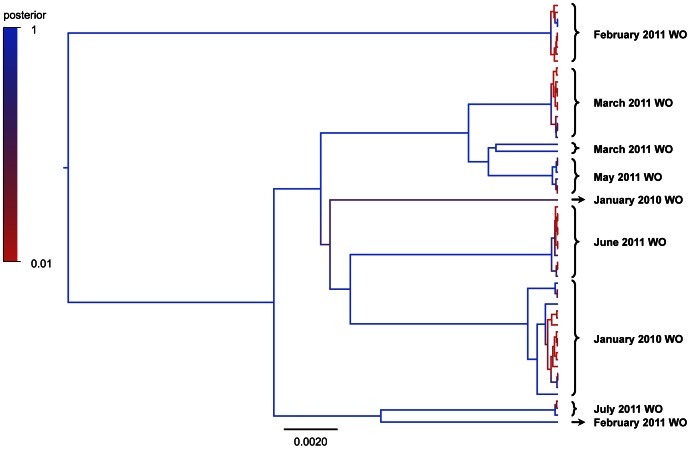
Evolutionary tree created by BEAST (Bayesian evolutionary analysis sampling trees) depicting all the full genomic sequences with relatedness (61 sequences, excluding repeated pairs). Clusters of genomes are visible among viruses sampled at similar points in time. Whole genome sequencing gives adequate resolution to distinguish potential divergent viral strains within the same time, as illustrated in clusters from January 2010, February 2011 and March 2011. WO = ward outbreak. Each node and branch has been coloured depicting the posterior probability supporting that clade calculated by Bayesian analysis (Dark Blue = 1 (high); Light Red = 0 (low)). Analysis was performed using BEAST v.1.7.5 combining two random number seed chains (10 million iterations each, saving 1 in 1000 iterations, with a 1 million iteration burn-in) using: HKY substitution; estimated frequency; strict clock; and constant population size coalescent tree prior. This maximum clade credibility tree was computed using TreeAnnotator v.1.7.5 and plotted with Figtree v.1.4.0.

We used BEAST [27] to reconstruct the evolutionary tree relating these 61 near-complete Norovirus genome sequences from patient samples (Figure 3). Viruses clustered in space and time, consistent with short-lived outbreaks, although divergent strains could be seen occurring within the same time period during ward outbreaks. Within spatio-temporal clusters, whole genome sequencing revealed low-level genetic variability (up to 13 SNVs). Between clusters, genomes diverged by up to 268 SNVs.

#### Improved fragmentation analysis

**Figure 4:**
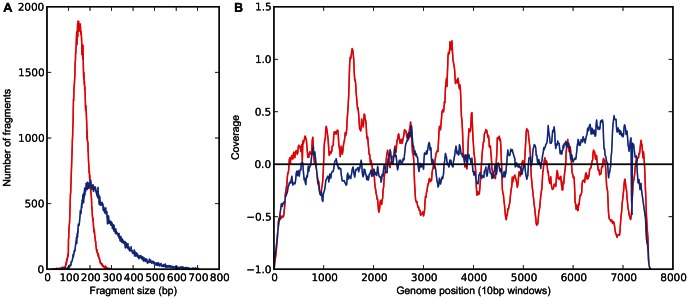
Comparison of the different fragmentation methods. A) fragment size distribution of a library prepared using the standard fragmentation method (red) and a library prepared using the new fragmentation method (blue). B) the coverage across the genome for the standard fragmentation sample (red) and the new fragmentation sample (blue). Data has been scaled as the difference from the median coverage for both samples.

Although the method proved to be robust, it may suffer from the limitation that high variation in coverage across the virus genome could require very large numbers of sequence reads to achieve acceptable sequence completeness (Figure S2). Since this could have arisen from interference of RNA secondary structures with efficient fragmentation, a modified fragmentation method was adopted that produced libraries with a wider distribution of insert sizes. This modification smoothed out coverage appreciably, improving data in those regions that previously were poorly represented (Figure 4). This improvement offers the prospect of increasing the throughput of samples from a single sequencing run.

## Discussion

Here we describe a reproducible next-generation short-read sequencing method based on a modified streamlined RNA-Seq approach for producing near-complete genome sequences of Norovirus and Hepatitis C virus. This method is scalable to batch processing of large numbers of samples with a relatively modest turn-around time of 4 weeks. The method is equally suited to rapid bench-top sequencing with a turn-around of under a week (Figure S3). The current cost of £60 per sample (consumables and sequencing) is similar to that achieved for sequencing a bacterial genome in our facility (Table 1). We have shown that in two distinct clinical sample types there is sufficient virus RNA present (as low as 0.01% or ∼4,200 reads mapping to that respective reference) to successfully undertake virus whole-genome sequencing. This is despite the presence of sequences from other sources such as human RNA and, in the case of faeces, food and microbial RNA. This improved method offers the prospect of large-scale affordable studies of RNA viruses and could be adapted to routine diagnostic laboratory workflows. An additional advantage of this method is that no step requiring sequence-specific primers for PCR amplification or bait-based enrichment is needed. This feature limits the possibility of primer-based bias in the processing of samples and provides the opportunity of bioinformatically detecting any other known RNA virus at the same time.

**Table 1:** A comparison of workflows and consumable costs for various viral sequencing approaches.

Method	Requires sequence specific primers/baits?	Failure Rate in Study (%)	Target Enrichment	mRNA Isolation	Material Fragmented	Size Selection	PCR cycles	Time per sample (days)	Sequencing Throughput (libraries/lane)	Consumable Cost per Sample
**RNA-Seq**	No	1/82 (1%)	N/A	PolyA Bead Isolation	mRNA to 150 bp	Yes	12	3.5 (same time up to 96 samples)	MiSeq = 6HiSeq = 96	£39
**Custom**	No	NA (test sample only)	N/A	N/A	Total RNA to 200 bp	N/A	12	3 (same time up to 96 samples)	MiSeq = 6HiSeq = 96	£35
**Sanger**	Yes	**39/55 (71%)**	Amplicon Generation	N/A	N/A	N/A	35	2	6–7	£47
**Amplicon-Seq**	Yes	71%*	Amplicon Generation	N/A	Amplicons to 400 bp	Yes	45	2	MiSeq = 96	£52
**SureSelect Hybridisation**	Yes	N/A	Probe Hybridisation	PolyA Bead Isolation	mRNA to 100 bp	N/A	10–14+12–16	5	MiSeq = 96	£270 probes only**
**Nugen Ovation**	No	N/A	N/A	N/A	N/A	N/A	12 ***	2.5	HiSeq = 96	£103 Ovation

The ‘Custom’ protocol refers to the modified RNA-Seq method used in this study to create larger insert fragments. Alternative methods include Amplicon-seq and hybridisation capture (SureSelect Target Enrichment for Illumina Paired-End mRNA-Seq Library Prep; version 1.1). Failure rates are determined by failure to sequence at least one amplicon (<86% of the genome). The failure rate for SureSelect is not given as it was not performed in our study. Consumable costs are list price per sample and exclude sequencing.

* failure rate based on that observed with Sanger Sequencing.

** estimated cost for probes only. Extra cost incurred for Agilent library preparation kit plus additional reagents recommended by Agilent.

*** linear amplification during SPIA reverse transcription has not been accounted for.

**Figure 5:**
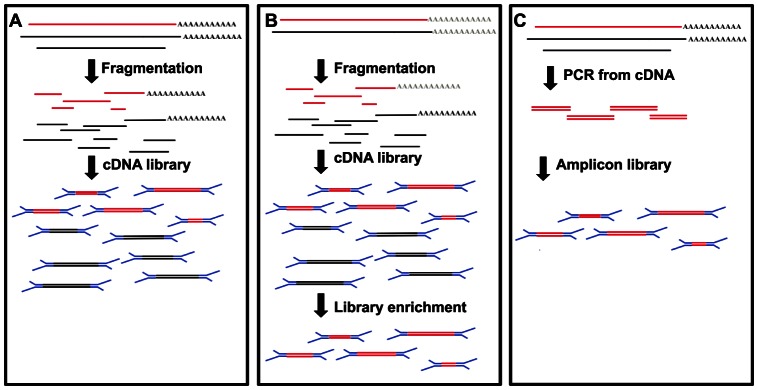
Schematic representation of different strategies for viral genome resequencing. A) Total RNA library: all the RNA species present in the sample are sequenced, no assumption on which genome is present, B) Hybridisation capture of a mRNA library: a good reference genome is needed to design the probes for capture, C) PCR enrichment: the desired genome is amplified from cDNA, a reference genome is needed to design specific oligos. Red lines, genomes of interest; Blue segments, Illumina adapters; Black lines, other RNA species.

Figure 5 shows a schematic representation of the various methods available for sequencing RNA viruses. We have shown that, for Norovirus, even for samples with a low titer (∼300 copies per microliter) it is possible to reconstruct near full genome sequence data (Table S2). However, in cases where the total available RNA is very limited and the viral load known to be ultra-low, other strategies may be implemented. As recently described the NuGEN Ovation RNA-Seq system provides a valid solution to amplify RNA viral genomes while removing rRNA [32]. It should be noted that this strategy adds to the cost of the preparation (Table 1). Moreover, the Single Primer Isothermal Amplification (SPIA) protocol relies on polyA degraded RNA species and non polyA containing species are expected to be lost or to only provide partial sequencing data.

One advantage of systems that can produce long cDNAs is the possibility of using the Illumina's Nextera kit which may greatly reduce the time for library construction. However, this is a speculative thought which has not been experimentally investigated. As such we prefer not to make hypothetical suggestions in the setting of this report.

Our RNA-Seq based method requires only 12 cycles of PCR amplification in total, while all amplicon or hybridisation based methods will include over 30 cycles of PCR (Table 1), increasing the likelihood of biases being introduced through amplification. We encountered a high failure rate with amplicon based methods, while our RNA-Seq based method was less susceptible to failures in reverse transcription, implying that the method may be more robust. The speed and throughput of the Illumina workflow make it a desirable approach for large numbers of samples (Figure S3), being able to prepare up to 96 samples in 3–3.5 days and sequencing them either in batches of 6 samples over 27 hours using the MiSeq or as a single batch of 96 over 14 days on a HiSeq.

The reproducibility of our sequencing method was demonstrated using multiple replicates of the same clinical samples (15 pairs) by the Illumina sequencing platform characterized by traditional Sanger sequencing. In the case of Norovirus, the sequences from strains associated with multiple temporally linked cases suggests that whole genomic sequence analysis is likely to provide a robust approach to tracking outbreaks. Furthermore, such high resolution data will also give rapid turnaround data on viral genotypes (e.g. HCV or HIV) and recognition of sudden shifts in the predominant capsid variant of Norovirus in general circulation [33] [34].

The depth of genome coverage enabled identification of within-sample sequence variation that probably reflects the presence of multiple, closely-related viral variants within the patient, sometimes known as “quasi-species” [35]. Analysis of our data revealed a small number of positions variable within individual Norovirus patients (up to 3 sites in one sample), which is the first evidence of low-level within-patient diversity in this virus. In contrast HCV exhibited a larger number of sites variable within individual patients (up to 131), consistent with previous reports [36]. Although mapping or assembly of short-read sequences obtained by next-generation sequencing has limited scope for reconstructing full-length virus haplotypes, useful insights may nevertheless be gained from the marginal frequency distribution of variants.

The success of our sequencing approach depends on bioinformatics methodology for recognising the viral sequences of interest as a minor component of a heterogeneous population of sequences. The mapping-based approach used here can be regarded as a prototype for characterizing viral genomes, and other strategies using assemblers of viral sequences may be more powerful. These include using a *de novo* population consensus assembly [37] [38] which may prove useful particularly when the virus population contains variation in genetic organisation such as large insertions and deletions. Current *de novo* assemblers have difficulty in robustly assembling virus genomes accurately in the presence of within-sample variability, contaminants, and variable sequence coverage [39]. However, new tools are becoming available which may combat these issues [40].

The improvements we describe in generating near whole viral genome sequences over sequences produced by Sanger sequencing [33] or Roche 454 sequencing [41] are likely to be superseded by future improvements in sequencing platforms, such as longer read and simplified sample preparation which requires lower input material [42]. It is expected that complete genome sequences may ultimately be obtained directly from clinical samples using these enhanced sequencing platforms and improved bioinformatics analysis in clinically relevant time-frames (e.g. within hours of receipt in a laboratory). These future methodologies may facilitate the discovery of new viruses once processing of all clinical samples in this way becomes routine practice. Such changes would revolutionise the diagnosis of viral infections and would also promote new avenues of research into virus evolution, antiviral resistance and personalized medicine approaches to treating specific viral genotypes.

## Supporting Information

Table S1 **Details of primers used for producing the seven overlapping amplicon fragments.** Internal primers have been included with the abbreviation INT followed by number. All primers listed were designed in-house. *F = forward; R = reverse.*
(DOCX)

Table S2 **Data generated by both Illumina platforms.** Samples with * in brackets denote samples that were originally sequenced using the MiSeq. Samples with alphabetical suffices (a and b) were pairs of samples re-sequenced from the same extraction of total RNA on the Illumina HiSeq to confirm reproducibility. Samples with Roman suffices (i and ii) denote pairs of samples where RNA extracted from the same faecal sample twice and two separate libraries prepared.
(XLS)

Figure S1 A comparison of the viral titre of each sample assessed by qPCR, and the percentage of reads obtained from each sample which mapped to the Norovirus reference genome, showing the correlation between the two measures (rho = 0.4, p<0.0001).
(TIFF)

Figure S2 **Coverage and percentage plots of 76 Norovirus HiSeq sequences.** A – percentage of the 76 Norovirus HiSeq samples which could be called at each position in the genome, averaged across 5 bp windows. B – the median, tenth percentile, and ninetieth percentile of the coverage across the genome for the 76 HiSeq samples. Note: 10^th^ percentile is 0 where not shown.
(EPS)

Figure S3 **Timelines associated with platforms per optimum batch size per lane.** Batch sizes are calculated by a combination of required coverage and the maximum multiplexing capacity available at the time. Sanger sequencing timeline does not take into account the high failure rate (71% in our study). PE = paired ends
(EPS)
