# Supplementary material for: A Modified RNA-Seq Approach for Whole Genome Sequencing of RNA Viruses from Faecal and Blood Samples
Source: PLoS One. 2013 Jun 10;8(6):e66129. doi: 10.1371/journal.pone.0066129 (PMC3677912; doi:10.1371/journal.pone.0066129)
Supplement: Table S1 — Details of primers used for producing the seven overlapping amplicon fragments. Internal primers have been included with the abbreviation INT followed by number. All primers listed were designed in-house. F = forward; R = reverse. (DOCX) [file pone.0066129.s001.docx]

**Table S1.** **Details of primers used for producing the seven overlapping amplicon fragments.**

| **Primer** | **Polarity** | **Sequence (5’-3’)** | **Binding site (nt)** |
| --- | --- | --- | --- |
| *PCR1F* | + | GTGAATGAAGATGGCGTCTAAC | 1 |
| *PCR1R* | - | GGGAARAACCAYTTCATGAC | 1160 |
| *INT1F* | + | CTCCCCAGTACCTCATCTC | 537 |
| *INT1R* | - | CCCAACAACAAAAGGCATAGC | 620 |
| *PCR-2F* | + | ATGTTGTCATCTGCTGCATCC | 1085 |
| *PCR-2R* | - | CACATTGCCCTTGCCATCAG | 2307 |
| *INT2F* | + | GAACTCGCTGACACTTGTCC | 1685 |
| *INT2R* | - | TGATAATGATGACATCGCTGTC | 1751 |
| *PCR3F* | + | GCTCAAGCAAGCACTCAAG | 2231 |
| *PCR3R* | - | AGGGACAGCCACAATCAC | 3438 |
| *INT3F* | + | AGAAGAGTACCTTCAGGACAG | 2758 |
| *INT3R* | - | CTCCTCCTCTTCACAGAAGTC | 2825 |
| *PCR4F* | + | CCCATGCAACCATGAAAATCC | 3336 |
| *PCR4R* | - | AGAGGGTGAGAAGCCAGTG | 4508 |
| *INT4F* | + | TGAGAAATGGTCGTTCGCAC | 3874 |
| *INT4R* | - | AGCTTGCCTGTGAAGGACTC | 3971 |
| *PCR5F* | + | TGGACGTGGGTGACTTCAC | 4425 |
| *PCR5R* | - | AGAAACTGTGAAGACATCGTCC | 5660 |
| *INT5F* | + | CAAGAGCCAATGTTCAGATGG | 5003 |
| *INT5R* | - | GTCACTCGACGCCATCTTC | 5087 |
| *PCR6F* | + | CTGTGTTGATCCCCTTACCCG | 5542 |
| *PCR6R* | - | GACGTCAGATGCCAATCCAG | 6726 |
| *INT6F* | + | CCCTCTAGGAACTCCAGATTTC | 6041 |
| *INT6R* | - | CTCCCAGTGTACACTGTAGC | 6129 |
| *PCR7F* | + | GTCAACCAGTTCTACACACTTGC | 6645 |
| *PCR7R* | - | NVTTTTTTTTTTTTTTTTTTTTTTTTTTTTTT | 7559 |
| *INT7F* | + | AGAGTTAAACAGGCAATGCTC | 6947 |
| *INT7R* | - | CTATTTTGATCCTCAACCCAGC | 7302 |

Internal primers have been included with the abbreviation INT followed by number. All primers listed were designed in house. *F= forward; R= reverse*
