# Supplementary figures and images for: A Modified RNA-Seq Approach for Whole Genome Sequencing of RNA Viruses from Faecal and Blood Samples
Source: PLoS One. 2013 Jun 10;8(6):e66129. doi: 10.1371/journal.pone.0066129 (PMC3677912; doi:10.1371/journal.pone.0066129)

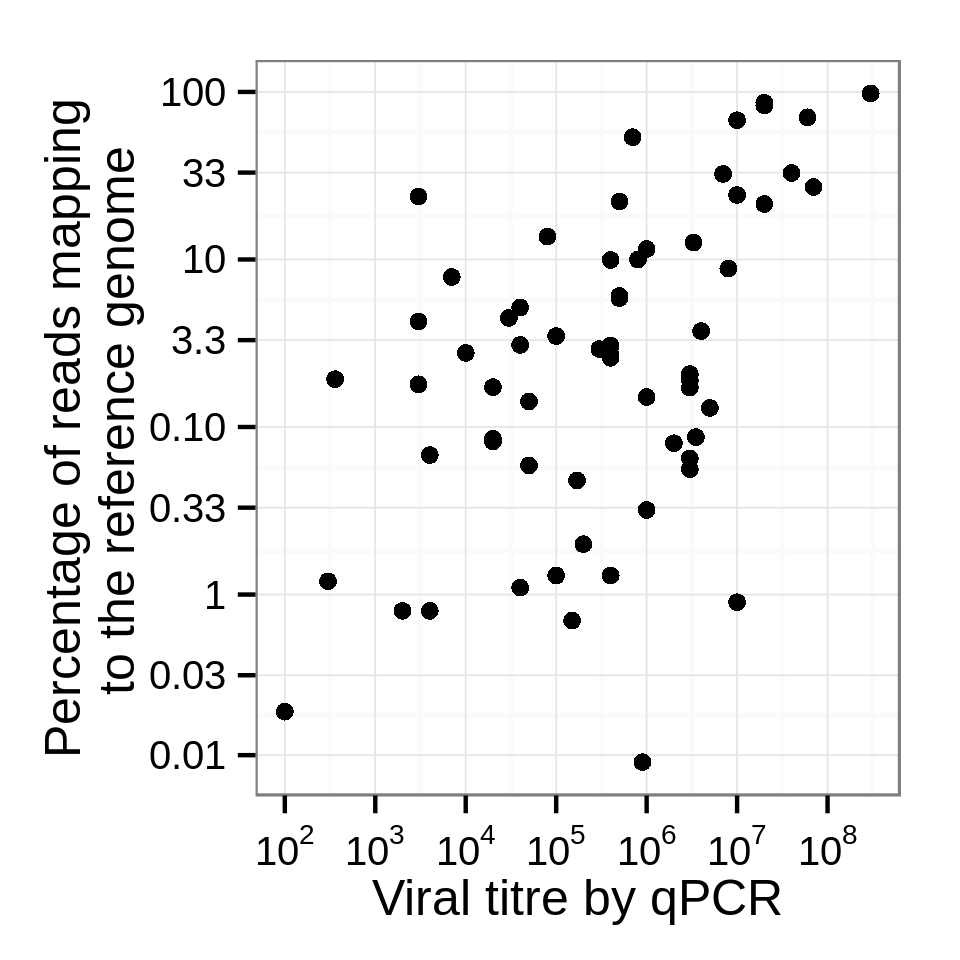

Supplement: Figure S1 — A comparison of the viral titre of each sample assessed by qPCR, and the percentage of reads obtained from each sample which mapped to the Norovirus reference genome, showing the correlation between the two measures (rho = 0.4, p<0.0001). (TIFF) [file pone.0066129.s003.tiff]
